# Differential metabolic and regulatory responses to exogenous glucose uptake across plant species revealed by isotopic metabolomics

**DOI:** 10.3389/fpls.2026.1872531

**Published:** 2026-08-04

**Authors:** Filipe Natalio

**Affiliations:** UCIBIO - Applied Molecular Biosciences Unit, Department of Chemistry, NOVA School of Science and Technology, University NOVA de Lisboa, Campus da Caparica, Caparica, Portugal

**Keywords:** *Arabidopsis thaliana*, *Gossypium hirsutum*, Micro-Tom, sugar uptake, untargeted metabolomic profiling, glucose

## Abstract

Plants rely on photosynthesis to fix carbon and produce sugars required for growth, energy metabolism, and structural development. The roots can also absorb exogenous sugars from the surrounding soil environment. However, how acquired sugars are metabolized, regulated, and integrated into plant metabolic networks, and how this varies across species, remains poorly understood. Untargeted metabolomic profiling was applied to below-ground and aerial tissues of *Arabidopsis thaliana*, *Solanum lycopersicum* cv. Micro-Tom, and *Gossypium hirsutum* seedlings treated with ¹²C or ¹³C_6_-glucose (30 mM, 48 h) under continuous illumination to minimize remobilization of endogenous carbon reserves. The ¹²C-metabolomic profiles revealed species-specific responses among the three species examined with 40 significantly altered metabolites in *A. thaliana* roots (predominantly decreased), 6 in *S. lycopersicum* roots (predominantly increased), and none in *G. hirsutum*. All three species transported ¹³C-glucose to aerial tissues, with *A. thaliana* showing the highest ¹³C enrichment (0.85% dry weight), compared to *S. lycopersicum* cv. Micro-Tom (0.20%) and *G. hirsutum* (0.34%). ¹³C-metabolomic profiling across root, hypocotyl, and shoot tissues identified species-specific sets of labeled metabolites in *A. thaliana*, *S. lycopersicum* cv. Micro-Tom, and *G. hirsutum*. Isotopologue distributions and the degree of correlation between tissues likewise differed among the three species, indicating divergent patterns of glucose-derived carbon partitioning. These findings demonstrate that while glucose uptake is conserved among these species, its metabolic impact and utilization are different among different plant species examined.

## 1 Introduction

Plants rely on photosynthesis to fix carbon and produce sugars required for growth, energy metabolism, and structural development. Roots, however, can also absorb exogenous sugars from the surrounding soil environment. This uptake of low molecular weight carbohydrates occurs within a highly competitive rhizosphere, where soil microorganisms depend on the same limiting substrates. It has been proposed that this capacity contributes to a carbon recovery mechanism that offsets losses associated with root exudation and rhizodeposition, through which plants release an estimated 1 to 10% of fixed carbon into the rhizosphere (Kuzyakov and Jones, 2006; Nguyen, 2003; Jones and Darrah, 1992; Jones et al., 2009). Despite this proposed role, how exogenously acquired sugars are metabolized, regulated, and incorporated into plant metabolic networks remains poorly understood.

Early physiological studies using radiolabeled sucrose and discs of mature sugar beet root tissue showed that sucrose uptake is energy-dependent, occurs against a concentration gradient, and involves carrier-mediated transport (Wyse, 1979). Further work in tissue culture systems further demonstrated that both autotrophic and heterotrophic plant cells can assimilate exogenous sugars, with uptake rates dependent on metabolic state and growth conditions (Wolf et al., 1998; Badr and Desjardins, 2007). For example, in potato plantlets, pulse-chase experiments with asymmetrically ¹^4^C-labeled sucrose showed uptake followed by hydrolysis, redistribution, and partial release of labeled glucose and fructose back into the medium, with stronger carbon turnover under carbon-limited conditions.

At the whole-plant scale, Kuzyakov and Jones (2006) (Kuzyakov and Jones, 2006) showed that ¹^4^C-glucose added to maize rhizosphere is rapidly cycled between soil, microbes, and plant tissues. Most of the label fraction was recovered in microbial biomass or respired as CO_2_, while only a small fraction was taken up by plants and an even smaller fraction was translocated to shoots. These results indicate again that root uptake of dissolved carbon occurs but is strongly limited by microbial competition in soil.

Furthermore, experiments in maize and cotton roots provided biochemical insight into the sugar uptake process, showing that glucose uptake and re-uptake are actively mediated by proton-coupled transport systems dependent on the plasma membrane electrochemical gradient. Disruption of this gradient or transporter activity using inhibitors such as carbonyl cyanide m-chlorophenyl hydrazone (CCCP), vanadate, or phlorizin reduces uptake and increases net efflux (Jones and Darrah, 1992, Jones and Darrah, 1993; Jones et al., 2009; Mühling et al., 1993). Also, it was demonstrated that members of the sugar transporter protein (STP) family mediate monosaccharide uptake in roots. For example, STP1 is mainly associated with constitutive uptake under normal conditions, while STP13 is induced under abiotic stress and contributes to the reabsorption of sugars released from damaged cells (Sacher, 1966; Yamada et al., 2011). STP13 is also induced during pathogen attack, where it reduces extracellular sugar availability and limits microbial access to carbon (Yamada et al., 2016).

Sugar uptake is not only related to carbon flux but also to activation of signaling pathways. For example, hexokinase 1 (HXK1)-dependent signaling connects intracellular sugar status with light and hormonal regulation, affecting gene expression and metabolic allocation, linking sugar signaling to transporter regulation under stress conditions (Koch, 1996; Moore et al., 2003; Rolland et al., 2006). During biotic stress, increased hexose-transporter activity reduces extracellular sugar availability, thereby limiting pathogen access to carbon, while under abiotic stress, such as salinity, altered transporter expression enhances the reabsorption of sugars released from damaged cells (Yamada et al., 2011, Yamada et al., 2016).

Despite the documented evidence of sugar uptake by roots, its metabolic impact and the metabolic chemical identity remain poorly understood. To address this, untargeted metabolomic profiling was performed on roots and shoots of wild-type (WT) *Arabidopsis thaliana*, *Solanum lycopersicum* cv. Micro-Tom, and *Gossypium hirsutum*, where the growth medium was supplemented with either ¹²C or ¹³C_6_-glucose under continuous illumination to prevent remobilization of endogenous carbon reserves. This approach allows regulatory responses, reflected in the ¹²C metabolome, to be distinguished from the fate of metabolized carbon, captured through ¹³C isotopologues. The ¹²C profiles revealed species-specific metabolic responses in roots and shoots for the examined species. All examined species transported ¹³C-glucose to aerial tissues, but isotopologue distributions and labeled metabolites differed substantially among these species, with minimal overlap.

## 2 Experimental procedure

### 2.1 Hydroponic growth of wild-type *Arabidopsis thaliana* (Col-0) seedlings

Wild-type *Arabidopsis thaliana* (Col-0) seeds were surface-sterilized by exposure to chlorine gas generated from 50 mL of 6% NaOCl and 1 mL of 32% HCl in a sealed container for 45 min at room temperature. Seeds were placed on open 1.5 mL microcentrifuge tube caps during sterilization. Following sterilization, seeds were sown onto modified 1.5 mL tube caps containing a central hole and filled with ½ Murashige and Skoog (MS) medium supplemented with 0.75% (w/v) agar. Seeds were stratified at 4 °C for 48 h. Caps containing seeds were then transferred to 6-well plates containing liquid ½ MS medium supplemented with 1% sucrose, with two seedlings per well. The caps were maintained floating on the medium surface. Subsequently, plates were moved to a growth chamber under long-day conditions (16 h light/8 h dark, 22 °C, 150 µmol m⁻² s⁻¹) and cultivated for 14 days in ½ MS medium supplemented with 1% sucrose. The medium was replaced with sugar-free ½ MS medium 48 h before any treatments.

### 2.2 Hydroponic growth of *Gossypium hirsutum*, and wild-type *Solanum lycopersicum* cv. Micro-Tom

Hydroponic growth of *Gossypium hirsutum* (Upland cotton) and wild-type *Solanum lycopersicum* cv. Micro-Tom was performed as follows. Seeds were surface-sterilized by immersion in 6% (v/v) NaOCl for 10–15 min, then rinsed thoroughly with sterile water. Sterilized seeds were germinated in closed 6-well plates on sterile filter paper moistened with ½ Murashige and Skoog (MS) medium. Germination was carried out in a growth chamber under controlled conditions (16 h light/8 h dark photoperiod, 22 °C, 150 µmol m⁻² s⁻¹) for 7 days. Seedlings were then transferred to a hydroponic system containing ½ MS medium supplemented with 1% sucrose/Plants were grown until the first true leaves were fully developed (~15 days) in greenhouse facilities under the following conditions: 25 °C day/19 °C night, ~50% relative humidity, and supplemental lighting from 06:00-10:00 and 16:00-19:00 to maintain adequate light intensity alongside natural daylight. The growth medium was replenished as needed, and the medium was replaced with sugar-free ½ MS medium 48 h before any treatments.

### 2.3 Feeding exogenous glucose to *A. thaliana*, wild-type *Solanum lycopersicum* cv. Micro-Tom and *Gossypium hirsutum*, and seedlings

Exogenous glucose feeding experiments were performed on hydroponically grown WT *Arabidopsis thaliana*, WT *Solanum lycopersicum* cv. Micro-Tom, and *Gossypium hirsutum* seedlings. Either unlabeled D-glucose (¹²C) or uniformly labeled ¹³C_6_ D-glucose (30 mM; Sigma-Aldrich, cat. no. 310808) was added to the growth medium. Control seedlings were maintained under identical conditions without glucose supplementation. For all treatments, seedlings were incubated for 48 h under continuous light at 25 °C. To avoid cross-contamination, control and ¹³C_6_-treated plants were maintained in separate containers. Following incubation, tissues were harvested as follows: roots and shoots from WT *A. thaliana*, and roots, hypocotyls, and first true leaves from *S. lycopersicum* and *G. hirsutum*. Roots were extensively rinsed with double-distilled water. For metabolomic analyses, parallel samples were immediately flash-frozen in liquid nitrogen and stored at -80 °C until processing. For isotope analysis, harvested tissues were dried at 60 °C for 48 h and analyzed using an Elemental Analyzer–Isotope Ratio Mass Spectrometer (EA-IRMS), as described below. All experiments were performed with four biological replicates.

### 2.4 Elemental analyser - isotope ratio mass spectrometer analysis

Carbon isotope composition was determined using an elemental analyzer-isotope ratio mass spectrometer (EA-IRMS) system consisting of a PDZ Europa ANCA-GSL elemental analyzer coupled to a Sercon 20–22 IRMS with SysCon electronics (SerCon, Cheshire, UK), operated at the Isotope Bioscience Laboratory, University of Ghent (Belgium). Isotope ratios were measured relative to laboratory reference materials and normalized to the VPDB scale. Calibration was performed using the following reference materials: WHEAT IA-R001 (δ¹³C_VPDB = −26.43 ± 0.08‰), Sorghum B-2159 (δ¹³C_VPDB = −13.78 ± 0.17‰), both calibrated against IAEA-CH6, and an in-house enriched maize reference material (δ¹³C_VPDB = 396.9 ± 3.28‰). Quality assurance (QA) samples were analyzed after every ten measurements. Analytical precision, assessed from five replicate measurements of a sorghum sample, yielded a standard deviation of 0.5‰. The standard deviation for the enriched QA reference was 0.8‰, with a mean deviation of 0.3‰. All statistical analyses were performed using JASP (v0.16.4) (Love et al., 2019).

### 2.5 Metabolomics

#### 2.5.1 Sample extraction

Metabolomics analysis was performed as follows. Approximately 100 mg of frozen plant tissue was weighed and transferred to 1.5 mL microcentrifuge tubes containing stainless steel beads. Samples were stored at −80 °C until extraction. Metabolites were extracted by adding 10× (v/w) methanol:water (80:20, v/v). Samples were homogenized using a bead-beater (4 × 30 s at 30 Hz) followed by ultrasonication for 5 min. Extracts were centrifuged at 18,000 × g for 5 min at 4 °C, and 500 µL of supernatant was collected and dried under a nitrogen stream. Dried extracts were reconstituted in 500 µL of eluent A1:B1 (1:1, v/v), vortexed for 30 s, centrifuged at 18,000 × g for 5 min at 4 °C, and filtered prior to analysis using spin filter tubes.

#### 2.5.2 Quality control samples

A pooled quality control (QC) sample was prepared by combining equal aliquots from all samples and injected at regular intervals throughout the analytical sequence to monitor instrument stability and analytical reproducibility.

#### 2.5.3 LC-MS method

Liquid chromatography-mass spectrometry (LC-MS) analysis was performed using a Vanquish UHPLC system coupled to a Q Exactive HF mass spectrometer (Thermo Fisher Scientific) equipped with an electrospray ionization source. Data were acquired in both positive and negative ionization modes using a previously described chromatographic method (Hsiao et al., 2018).

Raw data were processed using Compound Discoverer (v3.2, Thermo Fisher Scientific) and Skyline (Adams et al., 2020). Peak detection and initial integration were performed in Compound Discoverer, followed by manual curation in Skyline. Metabolite identification was assigned according to four confidence levels: Level 1 identification was based on matching retention time with authentic standards, accurate mass (≤3 ppm), and MS/MS spectra; Level 2a was assigned based on retention time and accurate mass (≤3 ppm); Level 2b was based on accurate mass (≤3 ppm) combined with MS/MS spectral matching; and Level 3 was based on accurate mass alone (≤3 ppm). QC samples were additionally analyzed in MS/MS mode to support compound annotation.

For ¹³C isotopic labeling experiments, compound detection and isotopologue analysis were performed using Compound Discoverer (v3.2). In addition to automated processing, manual targeted extraction was performed in Skyline using an in-house metabolite library. Data analysis followed a two-step workflow: initial untargeted identification using the full library, followed by targeted re-analysis of compounds not detected in the first step, including screening for potential ¹³C-labeled isotopologues. All samples were diluted fivefold before LC-MS analysis.

### 2.6 Pathway analysis

Pathway analysis was performed using log_2_ fold changes of statistically significant metabolites. Metabolites were mapped to pathways from KEGG Pathway Database () for *Arabidopsis thaliana* (ath), *Gossypium hirsutum* (ghi), and *Solanum lycopersicum* (sly). Network visualization and integration were conducted in Cytoscape (v3.9.1) (Shannon et al., 2003) using the KEGGscape plugin (Nishida et al., 2014). Relative intensities of ¹³C labeled metabolites were analyzed using an in-house Python script in two sequential steps: correction for natural ¹³C abundance, followed by estimation of inter tissue metabolite correlation based on Pearson correlation coefficients between datasets. The scripts are available from the corresponding author upon reasonable request.

### 2.7 Statistical analysis

Four biological replicates were analyzed per condition, tissue, and species. Statistical significance of metabolite abundance changes was assessed using a two-stage linear step-up procedure controlling the false discovery rate [p < (i/m)Q, Q = 0.05]. Given the number of metabolites assessed (174) across multiple tissues and species, this replicate number provides suitable sensitivity for detecting pronounced metabolic changes, though moderate effect sizes may be harder to resolve. Accordingly, the absence of statistically significant change should not necessarily be interpreted as evidence of biological absence of response.

## 3 Results

### 3.1 Untargeted metabolomic profiling of root and shoot tissues in WT *Arabidopsis thaliana*, WT *Solanum lycopersicum* cv. Micro-Tom, and *Gossypium hirsutum* seedlings treated with ¹²C D-glucose

#### 3.1.1 Wild-type *Arabidopsis thaliana* (Col-0)

**Figure 1:**
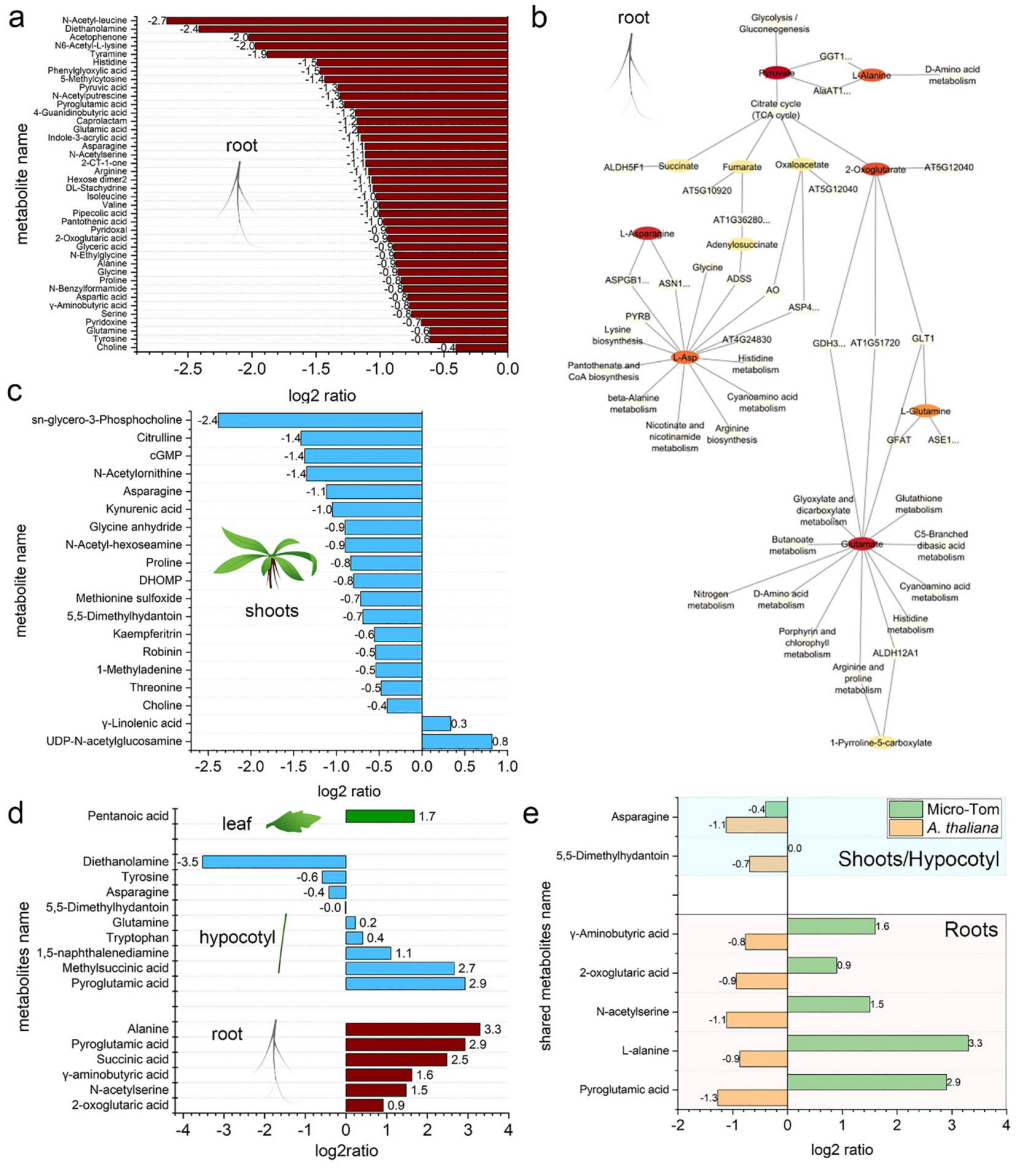
Untargeted metabolomic profiling of different tissues from WT *A. thaliana*, and roots and first true leaves of WT *Solanum lycopersicum* cv. Micro-Tom after feeding with exogenous ¹²C D-glucose for 48 h under continuous illumination. **(a)** Horizontal bar plot showing the log_2_ fold change (treatment/control) of 40 statistically significant metabolites [p < (i/m)Q, Q = 0.05] identified in roots of WT *A. thaliana*. Negative values indicate decreased metabolite abundance, whereas positive values indicate increased levels in response to ¹²C D-glucose treatment. Abbreviations: 2-CT-1-one, 2-(Cyclohexylmethylidene)-1,2,3,4-tetrahydronaphthalen-1-one; DHOMP, 2-(3,4-Dihydroxyphenyl)-5-hydroxy-4-oxo-4H-chromen-7-yl 6-O-(6-deoxy-α-L-mannopyranosyl)-β-D-glucopyranoside. **(b)** Pathway representation of selected statistically significant metabolites identified in *A. thaliana* roots. Metabolites are mapped onto their corresponding metabolic pathways, with log_2_ fold change values indicated by color coding, where orange represents the minimum value (-1.33), and yellow represents no change (0). **(c)** Horizontal bar plot showing the log_2_ fold change (treatment/control) of 20 statistically significant metabolites [p < (i/m)Q, Q = 0.05] identified in shoots of WT *A. thaliana*. **(d)** Horizontal bar plot showing log_2_ fold changes of 16 statistically significant metabolites (p < 0.05) identified in WT *Solanum lycopersicum* cv. Micro-Tom seedlings. Roots are shown in dark red, hypocotyls in light blue, and first true leaves in green. **(e)** Comparative analysis of commonly identified metabolites across tissues of WT *A. thaliana* (orange bars) and WT *Solanum lycopersicum* cv. Micro-Tom seedlings (green bars). Blue boxes indicate metabolites shared between WT *A. thaliana* shoots and WT *Solanum lycopersicum* cv. Micro-Tom hypocotyls, while light red boxes indicate metabolites shared between the roots of both species. The plot highlights contrasting metabolic responses to ¹²C D-glucose in roots versus aerial tissues.

Untargeted metabolomic profiling was performed independently on isolated roots and shoots of WT *Arabidopsis thaliana* (Col-0) growing hydroponically and after feeding with 30 mM ¹²C D-glucose for 48 h under continuous illumination. As a result, a total of 174 metabolites were putatively identified in control and glucose-treated samples. Principal component analysis (PCA) based on metabolites annotated at confidence levels 1, 2a, or 2b showed clear separation between treated and untreated roots (**Supplementary Figure 1**). In roots, 40 metabolites differed significantly between control and glucose-treated plants [p < (i/m)Q, Q = 0.05] (**Supplementary Table 1**), all showing decreased abundance after glucose treatment (**Figure 1a**), compared to untreated samples. Among these, pyruvate showed the highest decrease in abundance, followed by decreased downstream TCA cycle intermediates (fumarate, succinate, and 2-oxoglutarate), with 2-oxoglutarate showing the strongest decline. These changes were associated with reduced levels of several amino acids, including L-asparagine, L-glutamine, L-histidine, L-arginine, L-isoleucine, L-valine, and L-alanine (**Figure 1b**).

Pathway mapping of the significantly decreased root metabolites (**Figure 1b**) shows that pyruvate and its downstream TCA cycle intermediates (fumarate, oxaloacetate, and 2-oxoglutarate) act as central hubs connecting to a broad set of nitrogen-related pathways, including glutamate and glutamine metabolism, arginine and proline metabolism, histidine metabolism, and cyanoamino acid metabolism. This convergence indicates that glucose-induced remodeling in *A. thaliana* roots is not restricted to isolated amino acids but reflects a coordinated depletion of TCA cycle-derived carbon skeletons that supply multiple amino acid biosynthetic routes, consistent with reduced nitrogen assimilation and amino acid biosynthesis under exogenous glucose supply (Koch, 1996; Rolland et al., 2006).

As for shoots, PCA based on metabolites annotated at confidence levels 1, 2a, or 2b showed clear separation between treated and untreated shoots (**Supplementary Figure 1**). In shoots, 20 metabolites differed significantly between treatments [p < (i/m)Q, Q = 0.05], with most showing decreased abundance after glucose treatment (**Supplementary Table 2**). Exceptions included γ-linolenic acid and UDP-N-acetylglucosamine, which increased compared to untreated samples (**Figure 1c**). Affected metabolites were mainly associated with amino acid metabolism, including L-asparagine, L-threonine, L-proline, and the L-arginine precursors L-citrulline and N-acetylornithine. In addition, shoots showed reduced choline and sn-glycero-3-phosphocholine, indicating an effect on glycerophospholipid metabolism.

#### 3.1.2 Wild-type *S. lycopersicum* cv. Micro-Tom

Untargeted metabolomic profiling was performed independently on roots, hypocotyls, and first true leaves of WT *S. lycopersicum* cv. Micro-Tom seedlings growing hydroponically and after feeding with 30 mM ¹²C D-glucose for 48 h under continuous illumination. Also for this species, a total of 174 metabolites were putatively identified in control and glucose-treated samples. Principal component analysis (PCA), based on metabolites annotated at confidence levels 1, 2a, or 2b in the reduced dataset, showed clear separation among roots that were fed with glucose and those left untreated (**Supplementary Figure 1**).

In roots, six metabolites were significantly affected by glucose treatment [p < (i/m)Q, Q = 0.05] (**Supplementary Table 3**), all showing increased abundance: succinic acid, γ-aminobutyric acid, pyroglutamic acid, L-alanine, 2-oxoglutaric acid, and N-acetylserine (**Figure 1d**, red bars). Most of these metabolites are associated with alanine metabolism and the tricarboxylic acid (TCA) cycle.

As for hypocotyls, the PCA, based on metabolites annotated at confidence levels 1, 2a, or 2b in the reduced dataset, showed clear separation among hypocotyls that were fed with glucose and those left untreated (**Supplementary Figure 1**).

In hypocotyls, nine metabolites were significantly affected [p < (i/m)Q, Q = 0.05] (**Supplementary Table 4**). Pyroglutamic acid, methylsuccinic acid, 1,5-naphthalenediamine, L-tryptophan, L-glutamine, and 5,5-dimethylhydantoin increased in abundance, while L-asparagine, L-tyrosine, and diethanolamine decreased (**Figure 1d**, blue bars). Overall, glucose treatment was associated with increased abundance of amino acid-related intermediates in this tissue.

Finally, the PCA for metabolites annotated at confidence levels 1, 2a, or 2b in the reduced dataset identified in leaves also showed a clear separation (**Supplementary Figure 1**). However, in leaves, only pentanoic acid showed a significant increase compared to untreated samples (**Figure 1d**, green bar).

#### 3.1.3 Gossypium hirsutum

Untargeted metabolomic profiling was performed independently on roots, hypocotyls, and first true leaves of *Gossypium hirsutum* seedlings growing hydroponically and after feeding with 30 mM ¹²C D-glucose for 48 h under continuous illumination. In this case, a total of 174 metabolites were putatively identified in control and glucose-treated samples across tissues. The PCA for metabolites annotated at confidence levels 1, 2a, or 2b in the reduced dataset identified among different tissues showed a clear separation only for the cotton leaves. No metabolites showed statistically significant differences [p < (i/m)Q, Q = 0.05] in response to glucose treatment for roots, hypocotyls, and leaves.

### 3.2 ^13^C-labeled glucose feeding and ^13^C quantification in the upper tissues of all three species

To determine whether glucose fed to the roots of hydroponically grown seedlings of WT A*. thaliana*, WT *Solanum lycopersicum* cv. Micro-Tom, and *Gossypium hirsutum* was transported to aerial tissues, ¹³C-labeled glucose (¹³C_6_-Glc, 30 mM) was added to the growth medium. All seedlings were incubated for 48 h under continuous illumination to avoid dark-induced carbon allocation.

**Figure 2:**
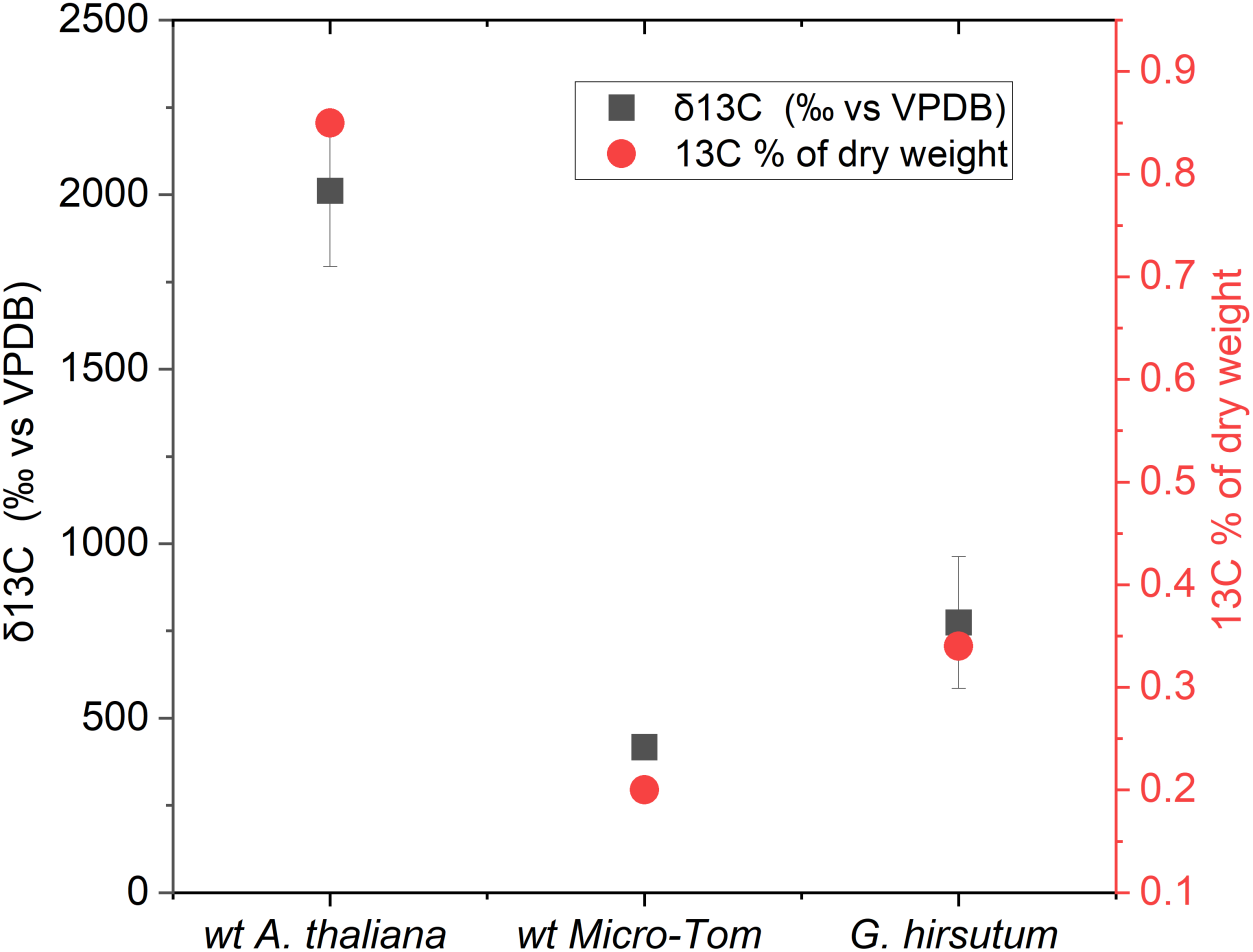
¹³C enrichment in aerial tissues of hydroponically grown seedlings following exogenous ¹³C_6_-glucose feeding. Comparative plot of background corrected average δ¹³C values (‰ vs. VPDB; left axis, grey squares) and ¹³C content (% w/w dry weight; right axis, red circles) measured in shoots or first true leaves of seedlings from three species: WT *A. thaliana*, WT *Solanum lycopersicum* cv. Micro-Tom, and *Gossypium hirsutum*. In all cases, ¹³C enrichment of aerial tissues was observed, although at different magnitudes across species. Seedlings were grown hydroponically and supplied with 30 mM ¹³C_6_-labelled glucose. Control plants were maintained under identical conditions without ¹³C_6_-glucose supplementation, reflecting natural ¹³C abundance. All experiments were conducted under continuous illumination for 48 h and in separate containers (see experimental section).

At the end of the experiment, roots and shoots of WT *A. thaliana*, and roots and first true leaves of WT *Solanum lycopersicum* cv. Micro-Tom and *G. hirsutum* were harvested and separated. For all three species, root tissues were not analyzed by elemental analyzer-isotope ratio mass spectrometry (EA-IRMS), as potential surface adsorption of ¹³C_6_-glucose could confound the resulting calculations. A comparative analysis of shoot tissues from three different seedlings revealed clear ¹³C enrichment across all three species (**Figure 2**), although with marked quantitative differences. WT *A. thaliana* shoots showed the highest enrichment, with a corrected mean δ¹³C value of 2011 ± 217‰ (**Figure 2**, grey squares), corresponding to approximately 0.85% ¹³C in dry weight (**Figure 2**, red circles). In comparison, leaves of WT *Solanum lycopersicum* cv. Micro-Tom and *G. hirsutum* exhibited lower enrichment, with δ¹³C values of 417 ± 13‰ and 774 ± 189‰, respectively (**Figure 2**, grey squares), corresponding to 0.20% and 0.34% ¹³C of dry weight (**Figure 2**, red circles). The variability was lowest in WT *Solanum lycopersicum* cv. Micro-Tom, as reflected by the small standard deviation, whereas *G. hirsutum* displayed greater dispersion in enrichment levels across biological replicates.

Overall, these results demonstrate that exogenously supplied ¹³C_6_-glucose is taken up by roots and transported to aerial tissues in all three species under hydroponic growth and continuous illumination over 48 h, resulting in measurable ¹³C accumulation in shoots and first true leaves. However, these measurements do not resolve the specific metabolic forms in which the carbon is transported or metabolized in these species.

### 3.3 ¹³C labeling analysis of isotopically correlated metabolite distribution across tissues

Untargeted metabolomic profiling of ¹³C-enriched metabolites was performed on tissues collected from seedlings of WT *A. thaliana*, WT *S. lycopersicum* cv. Micro-Tom, and *G. hirsutum* fed with 30 mM ¹³C_6_-glucose under hydroponic conditions for 48 h under continuous illumination. Profiling was conducted independently on roots and shoots of WT *A. thaliana*, and on roots, hypocotyls, and first true leaves of WT *S. lycopersicum* cv. Micro-Tom and *G. hirsutum*, with identified metabolites and their isotopologues corrected for natural ¹³C abundance prior to analysis. To assess relationships between tissue-specific metabolite profiles, Pearson correlation analysis was applied to ¹³C-labeled metabolite abundances across tissues. This correlation identifies statistical associations rather than evidence of metabolite movement or metabolic flux. Correlation thresholds of r ≥ 0.90, r ≥ 0.95, and r ≥ 0.99 were compared, and the lower thresholds retained substantially more metabolites, increasing the likelihood of spurious associations given the high dimensionality of the dataset. A conservative threshold of r ≥ 0.99 was therefore adopted to prioritize specificity, restricting downstream analysis to metabolite pairs with near-identical labeling trajectories between tissues. For WT *A. thaliana*, root and shoot profiles were correlated directly, whereas for WT *S. lycopersicum* cv. Micro-Tom and *G. hirsutum*, correlations were assessed first between roots and hypocotyls, and then between hypocotyls and first true leaves.

#### 3.3.1 Identification of ¹³C-labeled metabolites exhibiting correlated isotopologue patterns between tissues in WT *A. thaliana* seedlings fed with ¹³C_6_-Glc to the roots

**Figure 3:**
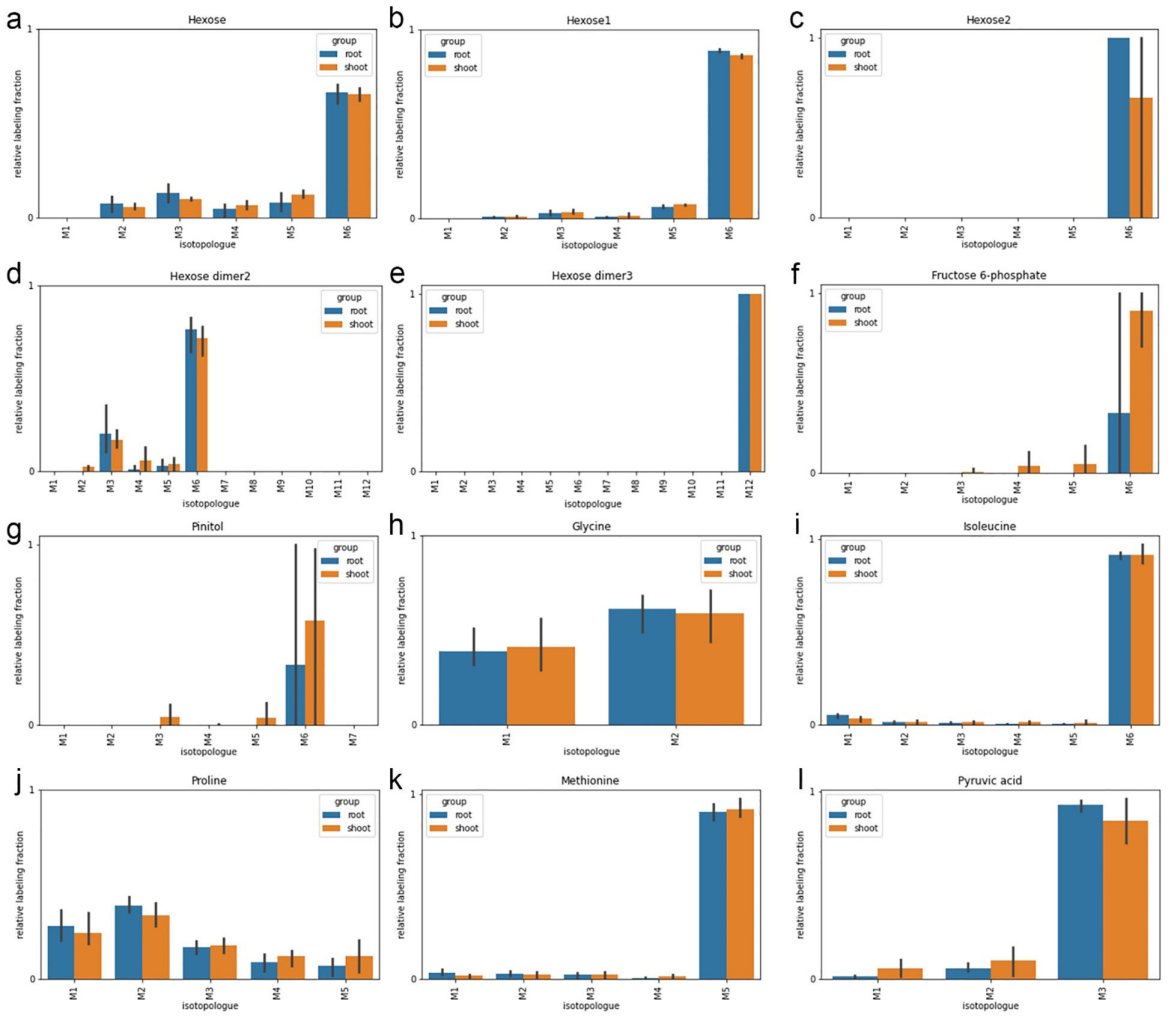
¹³C isotopologue distributions of correlated metabolites between roots and shoots in WT *A. thaliana* seedlings following exogenous ¹³C_6_-glucose feeding. Putatively identified metabolites with Pearson correlation ≥ 0.99 and their relative labeling fractions across isotopologues in roots and shoots of hydroponically grown *A. thaliana* seedlings supplied with 30 mM ¹³C_6_-glucose under continuous illumination for 48 h. M0 to MX denote isotopologues containing zero to X number ¹³C atoms per molecule, reflecting the number of labeled carbons incorporated from ¹³C_6_-glucose into each metabolite. **(a–g)**, sugar-related metabolites including hexoses, sugar phosphates, sugar alcohols, and disaccharides; **(h–k)**, amino acids (L-glycine, L-isoleucine, L-proline, and L-methionine); **(l)**, pyruvic acid (L-pyruvate).

In WT *A. thaliana*, 24 metabolites annotated at confidence levels 1 and 2a showed correlated labeling between roots and shoots (**Supplementary Table 5**), following the analysis described above. Shoots, hypocotyls and first true leaves were not separated and were therefore analyzed as pooled tissue. Sugar-related compounds comprised fructose-6-phosphate, cyclic sugar alcohol pinitol (Level 2a), hexose species (hexose, hexose1, hexose2), hexose dimers (hexose dimer2 and hexose dimer3), and disaccharide forms attributed to tagatose, glucose/galactose, inositol derivatives, melibiose (Level 1 to 2a depending on comparison), and cellobiose/maltose (**Figures 3a–g**). Amino acids included L-glycine, L-isoleucine, L-proline, and L-methionine (**Figures 3h–k**), together with taurine (Level 2a), lactate, and pyruvate (**Figure 3l**).

Hexose (tagatose) showed ¹³C incorporation across multiple isotopologues, with M6 as the dominant fraction (**Figure 3a**). Hexose1 (glucose/galactose) showed a similar pattern with stronger M6 enrichment and reduced intermediate isotopologues, while M1 remained unlabeled (**Figure 3b**). Hexose2 (inositol-related species) was mainly restricted to M6 (**Figure 3c**). Hexose dimer2 (melibiose) showed labelling up to M6 in shoots, with no signal from M7–M12 and a minor M12 contribution in roots; M6 and M3 formed the main labelled fractions (**Figure 3d**). Hexose dimer3 (cellobiose/maltose) was fully labelled with dominant M12 enrichment (**Figure 3e**), consistent with direct incorporation of ¹³C_6_-glucose units. Fructose-6-phosphate showed maximal M6 enrichment with higher relative labelling in shoots and minor M4–M5 contributions (**Figure 3f**). Pinitol also showed dominant M6 enrichment despite seven carbon atoms, with stronger labelling in shoots (**Figure 3g**).

Pyruvate and amino acids showed strong cross-tissue correlation and compound-specific isotopologue patterns (**Figures 3h–l**). L-glycine was mainly enriched in M1-M2, pyruvate, L-isoleucine, and L-methionine showed maximal enrichment at M3, M6, and M5, respectively, while L-proline displayed a broader distribution across isotopologues.

Overall, the 24 metabolites showed strong root-to-shoot correlation in their isotopologue patterns, covering sugars, amino acids, and organic acids. Sugars were generally dominated by M6 enrichment, whereas amino acids and organic acids displayed metabolite-specific isotopologue distributions ranging from low-carbon (M1-M2) to higher-order labeling (M3-M6).

#### 3.3.2 Identification of ¹³C labeled metabolites exhibiting correlated isotopologue patterns between tissues in WT *S. lycopersicum* cv. Micro-Tom seedlings fed with ¹³C_6_-Glc to the roots

**Figure 4:**
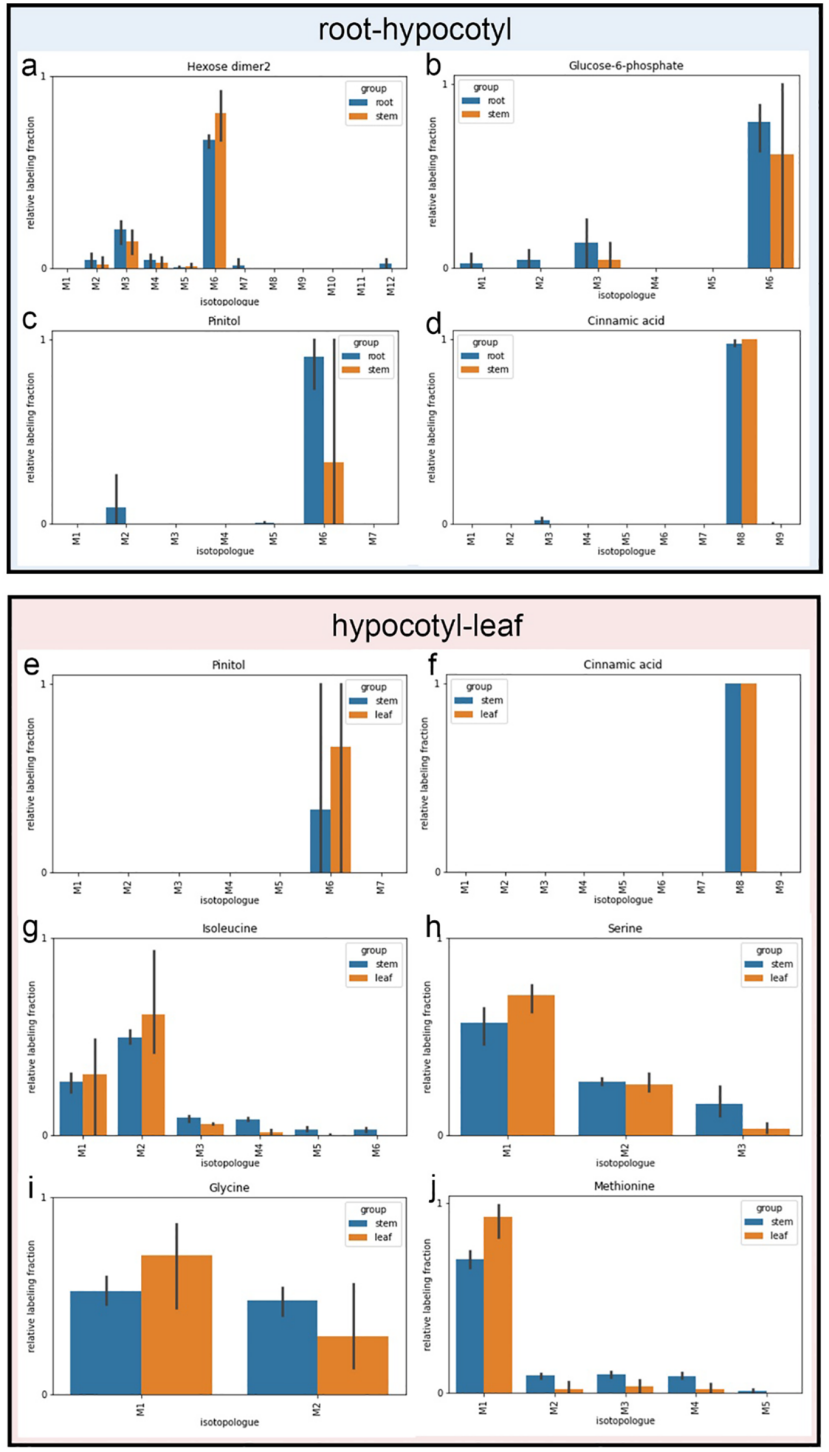
¹³C isotopologue distributions of correlated metabolites between root and hypocotyl, and between hypocotyl and leaves, in *S. lycopersicum* cv. Micro Tom seedlings following exogenous ¹³C_6_ glucose feeding. Putatively identified ¹³C enriched metabolites with Pearson correlation ≥ 0.99, their relative labeling fraction, and respective isotopologues between root and hypocotyl [blue box, **(a–d)**] and between hypocotyl and leaf [pink box, **(e–j)**] for wild type *S. lycopersicum* cv. Micro Tom seedlings growing hydroponically and fed exogenously with 30 mM ¹³C_6_ labeled Glc under continuous illumination for 48 h. The experiments were performed under continuous illumination for 48 h. M0 to MX denote isotopologues containing zero to X number ¹³C atoms per molecule, reflecting the number of labeled carbons incorporated from ¹³C_6_ glucose into each metabolite. **(a–d)**, root-hypocotyl metabolites putatively identified: hexose dimer 2 attributed to melibiose, glucose-6-phosphate, pinitol, and cinnamic acid. **(e–j)**, hypocotyl-leaves metabolites: pinitol, cinnamic acid, and the amino acids L isoleucine, L serine, L glycine, and L methionine.

In WT *S. lycopersicum* cv. Micro-Tom, 9 metabolites annotated at confidence levels 1 and 2a showed correlated labeling between roots and hypocotyl (**Supplementary Table 6**), following the analysis described above. These included hexose dimer2 (putatively attributed to melibiose; **Figure 4a**), glucose-6-phosphate (**Figure 4b**), pinitol (Level 2a; **Figure 4c**), cinnamic acid (Level 1 to 2a depending on comparison; **Figure 4d**), ferulic acid, uridine monophosphate (UMP), glycerol-3-phosphate, sn-glycero-3-phosphocholine, and taurine (Level 2a). Hexose dimer2 (melibiose) showed ¹³C enrichment up to the M6 isotopologue in hypocotyls, with no labeling from M7-M12, and a minor M12 signal in roots (**Figure 4a**). A secondary peak at M3 was also observed. Glucose-6-phosphate showed maximal enrichment at M6, followed by M3, with M1-M2 mainly restricted to roots (**Figure 4b**). Pinitol was likewise dominated by M6 labeling despite containing seven carbon atoms, with minor M2 enrichment in roots (**Figure 4c**). Cinnamic acid showed maximal enrichment at M8 rather than the expected M9, with only residual M3 labeling in roots (**Figure 4d**). Ferulic acid and related intermediates displayed lower but consistent labeling across tissues.

As for the hypocotyl-to-leaf, 11 metabolites annotated at confidence levels 1 and 2a showed correlated labeling (**Supplementary Table 7**), including pinitol, cinnamic acid, sugar phosphates, aromatic intermediates, and amino acids (**Figures 4e-j**). Pinitol and cinnamic acid were also correlated across the root-hypocotyl axis. Pinitol showed maximal M6 enrichment with higher labeling in leaves than hypocotyls, while cinnamic acid remained consistently enriched at M8 across all tissues. Amino acids showed predominantly low-carbon isotopologue enrichment, with L-isoleucine enriched mainly at M2 and L-serine, L-glycine, and L-methionine predominantly at M1.

Overall, the 9 root-hypocotyl and 11 hypocotyl-leaf correlated metabolites spanned sugars, aromatic compounds, and amino acids, with isotopologue patterns that were largely metabolite specific rather than uniform across compound classes. Sugar-related metabolites, including hexose dimer2 (melibiose), glucose-6-phosphate, and pinitol, were predominantly enriched at M6, while cinnamic acid showed consistent maximal enrichment at M8 across both tissue comparisons. Amino acids, in contrast, displayed predominantly low-carbon isotopologue enrichment (M1-M2), consistent with more restricted or diluted carbon incorporation relative to sugar-related compounds.

#### 3.3.3 Identification of ¹³C labeled metabolites exhibiting correlated isotopologue patterns between tissues in *Gossypium hirsutum* seedlings fed with ¹³C_6_-Glc to the roots

**Figure 5:**
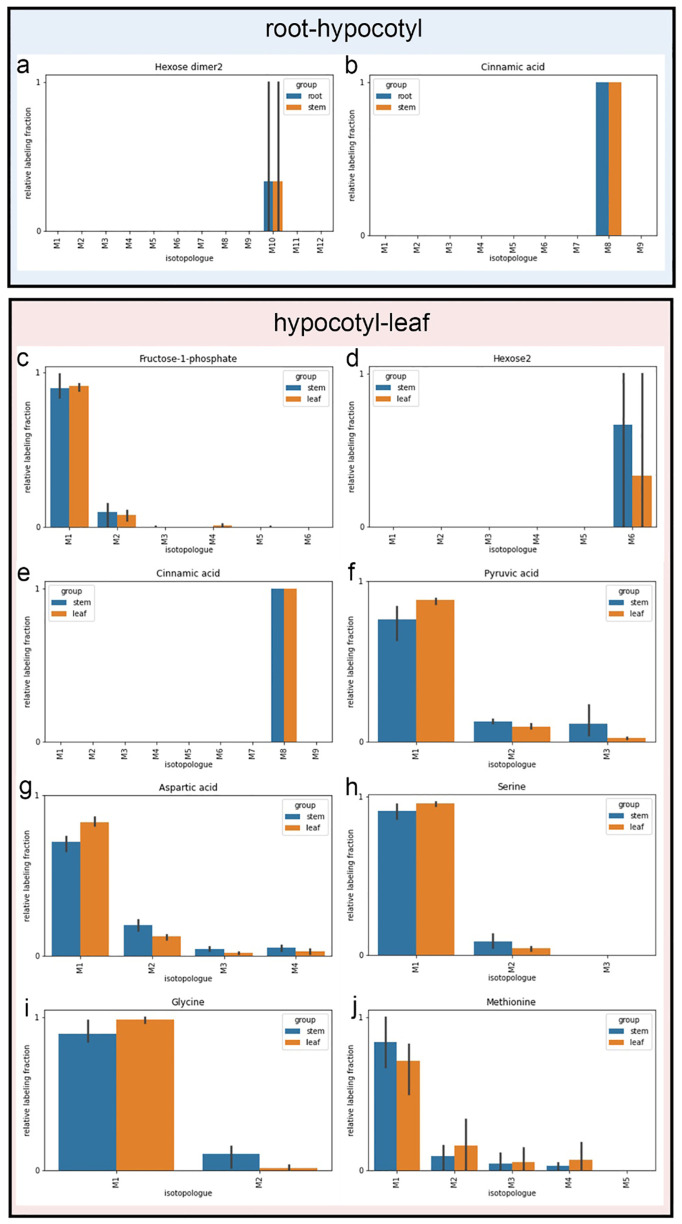
¹³C isotopologue distributions of correlated metabolites between root and hypocotyl, and between hypocotyl and leaves, in *G. hirsutum* seedlings following exogenous ¹³C_6_ glucose feeding. Putatively identified ¹³C enriched metabolites with Pearson correlation ≥ 0.99, their relative labeling fraction, and respective isotopologues between root and hypocotyl [blue box, **(a, b)**] and between hypocotyl and leaf [pink box, **(c–j)**] for *G. hirsutum* seedlings growing hydroponically and fed exogenously with 30 mM ¹³C_6_ labeled Glc under continuous illumination for 48 h. **(a, b)** identified root-hypocotyl metabolites: hexose dimer2 (attributed to melibiose) and cinnamic acid. **(c–j)**, hypocotyl-leaf metabolites: fructose-1-phosphate, hexose2, cinnamic acid, pyruvic acid, and amino acids L-aspartic acid, L-serine, L-glycine, and L-methionine.

In *G. hirsutum*, 8 metabolites annotated at confidence levels 1 and 2a showed correlated labeling between roots and hypocotyl (**Supplementary Table 8**), following the analysis described above. These included hexose dimer1 (unattributed), hexose dimer2 (melibiose; **Figure 5a**), cinnamic acid (**Figure 5b**), taurine, N-acetyl-hexosamine1, guanosine monophosphate (GMP), 4-pyridoxic acid, and 5-methoxysalicylic acid. Hexose dimer2 (melibiose) showed comparable relative labelling in roots and hypocotyls, but with a distinct isotopologue distribution relative to WT *A. thaliana* and WT *S. lycopersicum* cv. Micro-Tom, with enrichment mainly restricted to the M10 isotopologue (**Figure 5a**). Cinnamic acid was detected in both tissues with a consistent dominant M8 isotopologue, without additional minor labelling observed in WT *S. lycopersicum* cv. Micro-Tom roots (**Figure 5b**).

As for the hypocotyl-to-leaf, 19 metabolites annotated at confidence levels 1 and 2a showed correlated labeling. These include fructose-1-phosphate (**Figure 5c**), hexose2 (**Figure 5d**), hexose dimer1, aldopentose, cinnamic acid (**Figure 5e**), pyruvate (**Figure 5f**), ethylmalonic/methylsuccinic acid, lactate, and amino acids L-aspartate (**Figure 5g**), L-serine (**Figure 5h**), L-glycine (**Figure 5i**), and L-methionine (**Figure 5j**).

Fructose-1-phosphate showed maximal enrichment at M1, followed by M2. Hexose2 was exclusively enriched at M6, consistent with glucose/galactose assignment. Cinnamic acid maintained a stable dominant M8 isotopologue across all tissues. Pyruvate and amino acids were strongly correlated and mainly showed M1 enrichment, with generally higher M1 fractions in leaves than in hypocotyls for most compounds. Compared to WT *S. lycopersicum* cv. Micro-Tom, *G. hirsutum* showed reduced intermediate isotopologue contributions for L-serine, a higher M1 proportion for L-glycine, and a reduced leaf M1 fraction for L-methionine.

Overall, in *G. hirsutum*, 8 metabolites were associated with root-hypocotyl correlations and 19 with hypocotyl-leaf correlations, spanning sugars, sugar phosphates, aromatic intermediates, nucleotides, organic acids, and amino acids. Isotopologue patterns were highly constrained relative to the other two species, with several compounds dominated by a single isotopologue state, such as M10 for hexose dimer2 and M6 for hexose2, and cinnamic acid maintaining consistent M8 enrichment across tissues. Amino acids and central organic acids predominantly showed low-carbon enrichment, mainly restricted to M1.

#### 3.3.4 Identification and metabolite correlation across tissues and species and their metabolomic pathways

**Figure 6:**
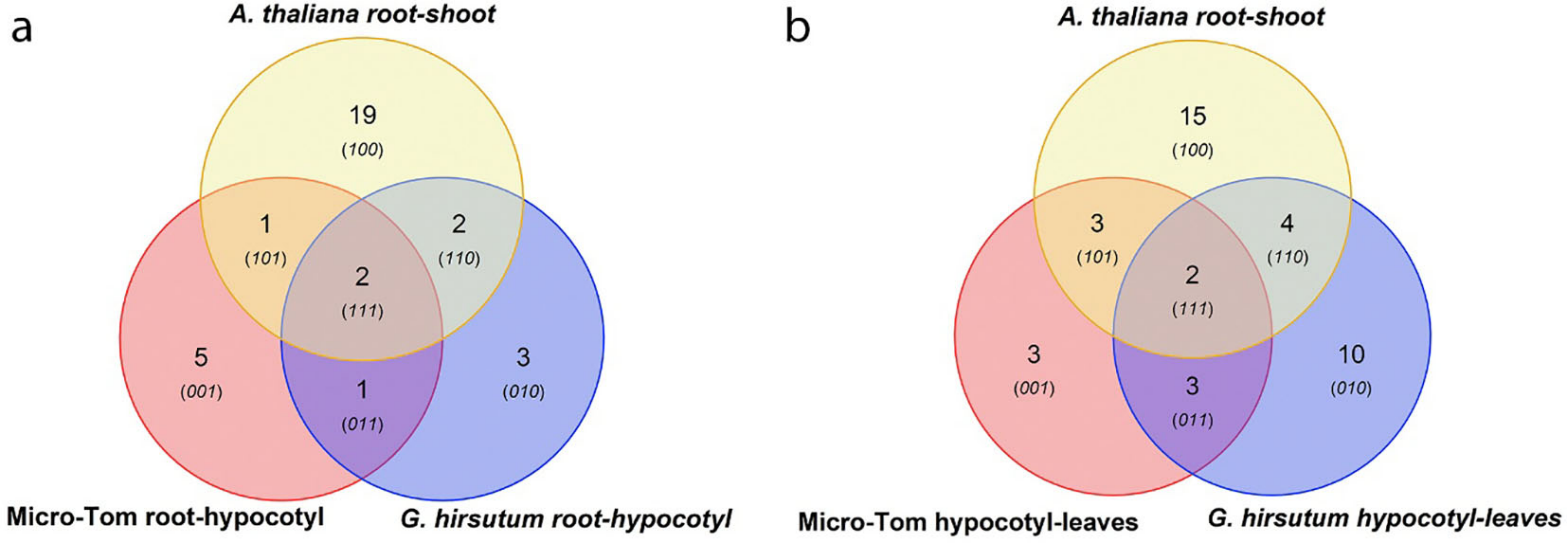
Venn diagrams of highly correlated putatively identified ¹³C-labelled metabolites (Pearson correlation ≥ 0.99) across *A. thaliana*, WT *S. lycopersicum* cv. Micro-Tom, and *G. hirsutum* across tissues. **(a)** Root shoot-hypocotyl correlations showing the distribution of shared and species-specific metabolites among the three species. This comparison highlights limited overlap across species, with most metabolites being species-specific, and identifies two metabolites common to all species: hexose dimer2 (melibiose) and taurine. **(b)** Shoot/hypocotyl-leaf correlations showing the distribution of shared and species-specific metabolites among the three species. Also, there is a limited overlap across species with only two metabolites being shared by all three species, namely, L-glycine and L-methionine.

A comparative analysis was performed across the examined species and tissues to evaluate the distribution of correlated ¹³C-enriched metabolites, along the root-shoot and hypocotyl-leaf (**Supplementary Table 10**). Among the 24 metabolites identified in root-shoot/hypocotyl comparisons of WT *A. thaliana*, 19 were species-specific (**Figure 6a**; **Supplementary Table 10**). Only pinitol was shared with WT *S. lycopersicum* cv. Micro-Tom, while five additional metabolites were shared with the other two species: N-acetyl-hexosamine1 and GMP with *G. hirsutum*, and ferulic acid, glucose-6-phosphate, glycerol-3-phosphate, sn-glycero-3-phosphocholine, and UMP with WT *S. lycopersicum* cv. Micro-Tom. In *G. hirsutum*, three metabolites were unique (hexose dimer1, 4-pyridoxic acid, and 5-methoxysalicylic acid). Cinnamic acid was shared between WT *S. lycopersicum* cv. Micro-Tom and *G. hirsutum*, while hexose dimer2 (melibiose) and taurine were common to all three species.

In the hypocotyl-leaf comparison, 15 metabolites were unique to WT *A. thaliana* (**Figure 6b**, **Supplementary Table 10**). Three metabolites were shared with WT *S. lycopersicum* cv. Micro-Tom (indole-3-carboxylate, L-isoleucine, pinitol) and four with *G. hirsutum* (hexose2, N-acetyl-hexosamine1, lactate, pyruvate). WT *S. lycopersicum* cv. Micro-Tom showed three species-specific metabolites (citraconic acid, pentose-5-phosphate, phosphoenolpyruvate), whereas *G. hirsutum* contained ten (including glycerate, trigonelline, adenine, hexose dimer1, indole, CMP, fructose-1-phosphate, ethylmalonic/methylsuccinic acid, L-aspartate, and nicotinamide). Aldopentose, cinnamic acid, and L-serine were shared between WT *S. lycopersicum* cv. Micro-Tom and *G. hirsutum*, while L-glycine and L-methionine were conserved across all three species.

Within the analyzed species, the metabolite overlap was limited. Root-shoot correlations within the analyzed species showed only hexose dimer2 (melibiose) and taurine as conserved metabolites. Hypocotyl-leaf comparisons also showed two metabolites overlapping, namely, pyruvate, hexose2, and lactate.

## 4 Discussion

The uptake of exogenous sugars such as glucose by roots has been documented as a physiological capacity involved in, for example, stress responses (Koch, 1996; Rolland et al., 2006; Yamada et al., 2011, Yamada et al., 2016). However, the metabolic responses and the extent to which exogenous glucose is metabolized and redistributed within the plant remained poorly understood. To address this gap, untargeted metabolomic profiling was applied to roots and shoots of three species representing a model dicot, a crop dicot, and a fiber crop, specifically, WT *Arabidopsis thaliana*, WT *Solanum lycopersicum* cv. Micro-Tom, and *Gossypium hirsutum* seedlings, which were supplied with either ¹²C or ¹³C_6_-labeled glucose. ¹²C-metabolomic profiles revealed how exogenous glucose alters the metabolic state, while the ¹³C-metabolomic profile showed the chemical forms in which glucose-derived carbon is metabolized to across different tissues.

Untargeted metabolomic profiling after ¹²C D-glucose exogenous feeding revealed differences in the number of statistically significant metabolite changes across the three species, from a pronounced response in WT *A. thaliana*, to an intermediate response in Micro-Tom, to no statistically significant changes detected in *G. hirsutum* under the conditions and sensitivity of this study. This gradient should be interpreted with caution, as factors such as biological variability, metabolite coverage, or dilution effects related to biomass differences could also contribute to the apparent absence of response in *G. hirsutum*. When these metabolites were introduced to the respective pathway, a pronounced reduction in TCA cycle intermediates and amino acids in WT *A. thaliana* was observed, suggesting that exogenous glucose may trigger a shift in central carbon allocation, possibly involving hexokinase-dependent glucose sensing (Koch, 1996; Rolland et al., 2006), a hypothesis that would require direct measurement of HXK1 activity or expression to confirm. In contrast, the accumulation of TCA intermediates and amino acids in *S. lycopersicum* roots could be consistent with enhanced nitrogen assimilation in response to exogenous glucose, although this interpretation remains hypothetical in the absence of direct nitrogen metabolism measurements. The absence of detectable change in *G. hirsutum* could indicate a reduced regulatory response to exogenous glucose in this species, or it may reflect biological variability, metabolite coverage, or dilution effects related to biomass differences or a combination of thereof.

The uptake of exogenous carbon-labeled sugars reported in this study is consistent with earlier observations in potato plantlets, maize, and cotton roots (Badr and Desjardins, 2007; Kuzyakov and Jones, 2006), expanding the number of plant species that display the capacity for sugar uptake by the roots. However, the variation in ¹³C enrichment between species differed substantially between the three studied’ species. This could be attributed to several factors that include biological variability, biomass differences, differential regulation of uptake, potentially involving members of the STP family (Yamada et al., 2011; Sacher, 1966; Yamada et al., 2016) and hexokinase-dependent signaling (Koch, 1996; Rolland et al., 2006). However, at this stage, it is not possible to decouple the role of each factor on ¹³C enrichment observed experimentally. Any mechanisms presented here remain hypothetical because transporter expression, enzyme activity, and gene expression were not measured in this study.

^12^C untargeted metabolite profiling of specific tissues reveals metabolic regulation as a response to glucose feeding, but it does not inform about chemical nature in which glucose is incorporated into downstream metabolism. A ¹³C-labelled metabolite profiling across different tissues was performed for the seedlings of the three plant species to address this question. Then, Pearson correlation analysis was applied to ¹³C-labelled metabolite abundances across tissues to explore relationships between tissue-specific metabolite profiles, where a conservative threshold of r ≥ 0.99 was adopted to prioritize specificity. However, it is worth noting that this correlation identifies statistical associations rather than evidence of metabolite movement or metabolic flux (Krumsiek et al., 2011; Steuer et al., 2006; Ratcliffe and Shachar‐Hill, 2006). Pinitol, taurine, and cinnamic acid were identified at confidence Level 2a based on accurate mass and retention time without authentic standard confirmation, and melibiose identification confidence ranged from Level 1 to Level 2a depending on the tissue comparison. Thus, any conclusions involving these metabolites should be interpreted cautiously until confirmed with authentic standards.

Nevertheless, this approach revealed a limited subset of ¹³C-labelled metabolites whose abundances were strongly correlated between tissues. The identified metabolites included sugars, amino acids, and organic acids, but their number and identity differed substantially across species, with only a small fraction conserved. This indicates that, although glucose uptake and metabolization occur across species, the allocation of glucose-derived carbon into specific metabolite pools is specific for the examined species. This notion was further supported by the species-specific isotopologue patterns analysis, labelling distributions, and degrees of carbon rearrangement. These results are consistent with differential routing of carbon through primary and secondary metabolic pathways in plants (Ratcliffe and Shachar‐Hill, 2006; Schwender et al., 2004; Allen and Young, 2020). Together, these results indicate that while the capacity for exogenous glucose uptake and transport is conserved for the examined species, its downstream metabolism and the pattern of isotopic correlation across tissues vary between them. However, interspecies comparisons should be interpreted with caution. *A. thaliana* was grown under controlled growth chamber conditions, whereas *S. lycopersicum* cv. Micro-Tom and *G. hirsutum* were grown under greenhouse conditions, introducing differences in light, temperature stability, and humidity. In addition, the tissue sets differ between species: root and shoot for *A. thaliana* versus root, hypocotyl, and leaf for *S. lycopersicum* and *G. hirsutum*, and the species differ in developmental stage, morphology, and biomass at the time of sampling. These factors may contribute to the observed differences in metabolic response and should be considered when interpreting species-specific conclusions. Yet, the subtle metabolic changes may have remained undetected in *G. hirsutum* seedlings and could be attributed to statistical power, low number of replicates, and the use of a single concentration of glucose.

Still, a common experimental setup across all three plant species was that, during ^12^C and ^13^C glucose feeding, continuous illumination was used to prevent dark-induced remobilization of endogenous carbohydrates, which would otherwise obscure the metabolic signatures of exogenous glucose (Sulpice et al., 2009; Gibon et al., 2004; Graf et al., 2010). Also, the use of a hydroponic system provided controlled conditions free from soil heterogeneity and microbial competition, enabling interferences and creating additional difficulties in data interpretation (Kuzyakov and Jones, 2006; Nguyen et al., 2016). However, these controlled conditions remove key ecological constraints such as circadian dynamics, microbial interactions, and soil composition variability that would be present under field conditions. Thus, these results reflect intrinsic plant metabolic capacity under standardized conditions rather than realistic agronomic scenarios, and should be interpreted accordingly.

From a biosynthetic standpoint, the isotopologue distributions observed for these analyzed species are consistent with expected carbon routing. Sugars and sugar derivatives were predominantly M6 labeled in both species, consistent with direct incorporation of the intact ¹³C_6_ glucose backbone with minimal carbon loss. Pyruvate showed predominantly M3 labeling, matching the transfer of an intact three-carbon unit from glycolytic cleavage of labeled glucose. Amino acids showed more fragmented, lower mass isotopologue distributions (M1 to M3), consistent with carbon skeleton turnover through glycolysis and the TCA cycle prior to incorporation into amino acid backbones. Pinitol and cinnamic acid, which arise through more complex, multi-step biosynthetic routes, showed intermediate and mixed labeling patterns, in line with their more indirect derivation from glucose-derived carbon.

Among the metabolites overlapping for these analyzed species, hexose dimer2 (tentatively identified as melibiose) was one of only two metabolites shared in the root-shoot/root-hypocotyl comparison across all three species. Yet, they show distinct isotopologue distributions between them. In *A. thaliana* root and shoot, and in *S. lycopersicum* cv. Micro-Tom root and hypocotyl, hexose dimer2 was dominated by the M6 isotopologue, with a smaller, lower mass shoulder (M2 to M3) also present in both tissues. This lower mass fraction indicates that, in addition to direct condensation of a labeled hexose unit, a portion of the pool is remodeled through partial carbon exchange, consistent with a metabolic network subject to feedback regulation. In *A. thaliana*, this is further supported by the related disaccharide hexose dimer3, which showed complete M12 labeling, indicating condensation of two intact labeled hexose units. In contrast, *G. hirsutum* showed a distinct dominant isotopologue for hexose dimer2, M10, differing from the M6 pattern observed in the other two species.

A final key question arising from these findings is whether exogenous glucose is likely to be encountered by roots under realistic soil conditions. In agricultural soils, soluble carbohydrates are continuously generated through root exudation, microbial turnover, and the decomposition of plant residues, contributing to the dissolved organic carbon pool in the rhizosphere (Jones and Darrah, 1992). Within this pool, monosaccharides such as glucose are present in concentrations in the low millimolar to sub-millimolar range, although their concentrations fluctuate strongly with soil type, moisture, and microbial activity (Jones and Darrah, 1992; Nguyen, 2003). This creates a highly dynamic environment in which simple sugars are available but rapidly used, exudated, and recycled (Kuzyakov and Jones, 2006). The glucose concentration applied in this study exceeds typical bulk soil concentrations and was selected to ensure detectable isotopic enrichment rather than to approximate a specific natural exposure level. Localized and transient glucose concentrations in the rhizosphere, for example following root cell lysis, root exudation bursts, or microbial turnover, may transiently approach or exceed this range, but this should not be interpreted as representative of steady-state field conditions (Jones and Darrah, 1992; Kuzyakov and Jones, 2006; Nguyen, 2003). Overall, the results should be interpreted as describing the intrinsic capacity of these particular species to uptake, transport, and metabolize glucose under controlled exposure conditions and should not be extrapolated to ecological or field-relevant scenarios. Whether these metabolic changes translate into altered growth, stress tolerance, or carbon economy remains untested and represents an important direction for future studies.

## 5 Conclusion

In conclusion, this work shows that *Arabidopsis thaliana*, *Solanum lycopersicum* cv. Micro-Tom, and *Gossypium hirsutum* can uptake glucose given exogenously by the roots, transported to aerial parts, and metabolized in different tissues. Although this capacity was observed in all three species, the extent of ^13^C enrichment, isotopologue distributions, and metabolite profiles differed substantially, suggesting species-specific regulation of glucose-derived carbon allocation and metabolism for these species in particular. These findings describe intrinsic physiological capacity under standardized experimental conditions for these three species and should not be directly extrapolated to any ecological or field-relevant scenarios. Future studies would aim to investigate glucose uptake, carbon partitioning, and metabolic fate in mature plants grown under both hydroponic and soil-based conditions to assess the relevance of these processes under more realistic environmental conditions.

## Data Availability

The original contributions presented in the study are included in the article/**Supplementary Material**, further inquiries can be directed to the corresponding author.
