# Supplementary material for: Differential metabolic and regulatory responses to exogenous glucose uptake across plant species revealed by isotopic metabolomics
Source: Front Plant Sci. 2026 Aug 4;17:1872531. doi: 10.3389/fpls.2026.1872531 (PMC13481508; doi:10.3389/fpls.2026.1872531)
Supplement: Supplementary file 1 [file Supplementaryfile1.pdf]

Differential metabolic and regulatory responses to exogenous glucose uptake across plant species revealed by isotopic metabolomics

Filipe Natalio[1*]

[1] UCIBIO – Applied Molecular Biosciences Unit, Department of Chemistry, NOVA School of Science and Technology, University NOVA de Lisboa, Campus da Caparica, Caparica, Portugal

*Correspondence should be addressed to: filipe.natalio@fct.unl.pt

Keywords

Gossypium hirsutum, *Arabidopsis thaliana,* Micro-Tom, sugar uptake, untargeted metabolomic profiling

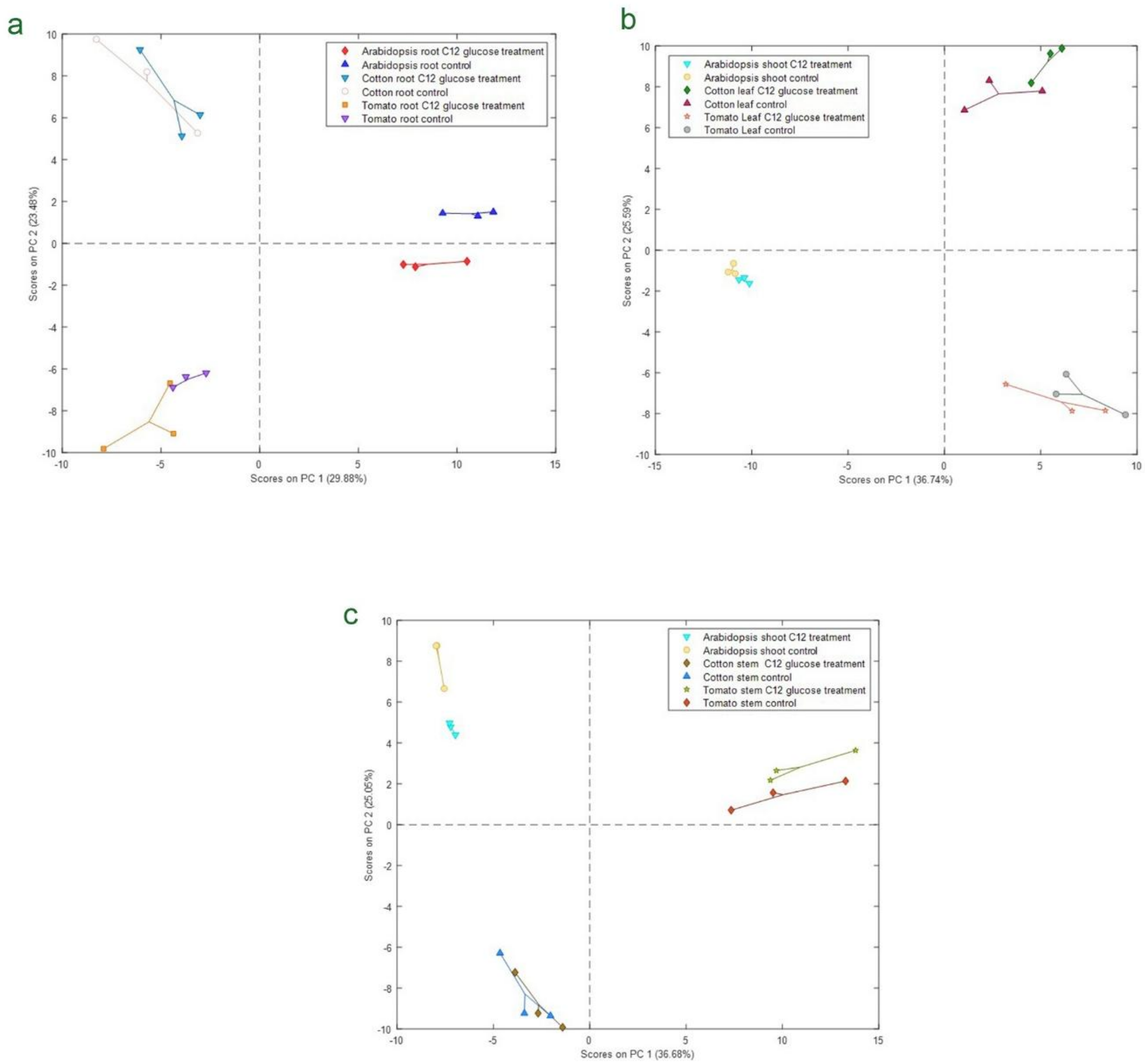

Figure S1. Principal component analysis (PCA) of untargeted metabolomic profiles from WT *A. thaliana*, WT *S. lycopersicum* cv. Micro-Tom, and *G. hirsutum* seedlings after feeding with ^{12}C D-glucose or control for 48 h under continuous illumination, based on metabolites annotated at confidence levels 1, 2a, or 2b. (a) Root. (b) Leaf/shoot. (c) Stem/hypocotyl. In all three tissues, PCA scores cluster primarily by species, with control and ^{12}C glucose-treated replicates overlapping within each species cluster and no consistent separation attributable to treatment.

Table S1. List of the intensity levels, log2 ratio, and level of annotation of the statistically significant metabolites ($p<0.05$) found in the **roots** of WT *A. thaliana* after treatment with ^{12}C D-glucose vs. control for 48h under continuous illumination.

metabolite name	Control1	Control2	Control3	^{12}C Glc_1	^{12}C Glc_2	^{12}C Glc_3	log2 ratio	level of annotation
5-Methylcytosine	1.34E+06	1.45E+06	1.31E+06	5.78E+05	4.76E+05	4.66E+05	-1.4	2b
Asparagine	1.87E+08	1.66E+08	1.81E+08	8.57E+07	7.98E+07	8.11E+07	-1.1	1
Hexose dimer2	4.02E+06	4.26E+06	3.94E+06	2.17E+06	1.89E+06	1.78E+06	-1.1	1
Histidine	7.04E+07	6.98E+07	7.15E+07	3.15E+07	2.21E+07	2.17E+07	-1.5	2a
Acetophenone	5.05E+06	4.50E+06	4.42E+06	1.45E+06	1.05E+06	9.36E+05	-2.0	2b
Tyramine	2.33E+06	2.01E+06	2.12E+06	7.10E+05	5.56E+05	4.87E+05	-1.9	2b
Pyroglutamic acid	1.67E+08	1.56E+08	1.80E+08	7.43E+07	6.95E+07	6.39E+07	-1.3	2a
N-Acetyl-leucine	9.45E+04	8.19E+04	8.16E+04	4.68E+03	2.03E+04	1.57E+04	-2.7	2a
Glycine	8.66E+06	8.79E+06	7.76E+06	4.64E+06	4.46E+06	4.79E+06	-0.9	2a
Serine	5.67E+07	5.51E+07	5.89E+07	3.03E+07	3.53E+07	3.57E+07	-0.8	1
Indole-3-acrylic acid	6.73E+06	7.45E+06	7.94E+06	3.54E+06	3.42E+06	3.02E+06	-1.1	1
Arginine	9.12E+07	9.77E+07	1.09E+08	4.97E+07	4.50E+07	4.51E+07	-1.1	1
2-(Cyclohexylmethylidene)-1,2,3,4-tetrahydronaphthalen-1-one	7.69E+06	8.91E+06	7.54E+06	3.88E+06	3.53E+06	3.75E+06	-1.1	1
N-Benzylformamide	2.71E+06	2.69E+06	2.48E+06	1.29E+06	1.54E+06	1.63E+06	-0.8	2b
Alanine	5.20E+07	5.64E+07	5.78E+07	3.48E+07	2.80E+07	2.79E+07	-0.9	2a
DL-Stachydrine	3.29E+06	3.37E+06	2.76E+06	3.54E+06	1.63E+06	1.52E+06	-1.1	2b
Caprolactam	4.70E+06	5.05E+06	4.91E+06	2.81E+06	1.71E+06	1.96E+06	-1.2	2b
N-Acetylputrescine	3.75E+06	4.31E+06	4.12E+06	2.12E+06	1.19E+06	1.59E+06	-1.3	2b
Aspartic acid	2.62E+07	2.37E+07	2.61E+07	1.70E+07	1.39E+07	1.32E+07	-0.8	2a
Pantothenic acid	6.73E+06	6.98E+06	7.03E+06	2.68E+06	3.58E+06	4.30E+06	-1.0	2a
4-Guanidinobutyric acid	2.23E+06	1.94E+06	1.70E+06	8.68E+05	8.06E+05	8.98E+05	-1.2	2a
Glutamic acid	1.32E+07	1.13E+07	1.01E+07	5.70E+06	4.92E+06	4.72E+06	-1.2	1
N-Acetylserine	1.08E+07	1.20E+07	1.09E+07	3.66E+06	6.40E+06	5.55E+06	-1.1	1
Glyceric acid	4.71E+07	5.01E+07	5.78E+07	3.12E+07	2.67E+07	2.54E+07	-0.9	1
N-Ethylglycine	2.63E+06	1.87E+06	5.89E+06	5.53E+05	7.51E+05	3.11E+06	-0.9	2b
Pyridoxine	2.81E+07	3.24E+07	3.28E+07	1.81E+07	2.17E+07	1.86E+07	-0.7	1
Choline	6.43E+09	6.88E+09	6.40E+09	5.36E+09	4.85E+09	4.67E+09	-0.4	2b
Valine	9.19E+07	1.05E+08	1.09E+08	6.38E+07	4.54E+07	4.29E+07	-1.0	2a
2-Oxoglutaric acid	2.35E+06	2.39E+06	2.37E+06	8.70E+05	1.46E+06	1.39E+06	-0.9	2a
γ-Aminobutyric acid	4.44E+08	4.56E+08	4.96E+08	3.29E+08	2.37E+08	2.53E+08	-0.8	1
Proline	2.10E+08	2.14E+08	2.13E+08	1.51E+08	1.01E+08	1.05E+08	-0.8	1

Isoleucine	8.57E+07	1.02E+08	1.02E+08	6.15E+07	4.09E+07	3.99E+07	-1.0	1
Phenylglyoxylic acid	1.60E+05	1.28E+05	1.59E+05	2.63E+04	6.26E+04	7.26E+04	-1.5	2a
Diethanolamine	7.37E+08	1.08E+09	7.90E+08	3.43E+08	8.63E+07	5.86E+07	-2.4	2b
Pyruvic acid	1.03E+07	8.11E+06	7.50E+06	4.57E+06	3.16E+06	2.59E+06	-1.3	2a
N6-Acetyl-L-lysine	6.09E+05	5.37E+05	7.15E+05	2.37E+05	2.77E+03	2.33E+05	-2.0	2b
Glutamine	1.06E+09	9.46E+08	1.18E+09	6.55E+08	7.69E+08	6.58E+08	-0.6	1
Tyrosine	2.84E+07	3.28E+07	3.23E+07	2.42E+07	1.88E+07	1.83E+07	-0.6	1
Pipecolic acid	2.47E+06	2.30E+06	2.17E+06	1.30E+06	1.47E+06	6.74E+05	-1.0	2b
Pyridoxal	2.75E+07	3.11E+07	2.47E+07	1.90E+07	1.26E+07	1.15E+07	-0.9	2a

Table S2. List of the intensity levels, log2 ratio, and level of annotation of the statistically significant metabolites ($p<0.05$) found in the **shoots** of WT *A. thaliana* after treatment with ^{12}C D-glucose vs. control. for 48h under continuous illumination.

metabolite name	Control1	Control2	Control3	^{12}C Glc_1	^{12}C Glc_2	^{12}C Glc_3	log2 ratio	level of annotation
N-Acetyl-hexoseamine	7.52E+06	9.55E+06	6.33E+06	4.45E+06	5.21E+06	6.02E+06	-0.9	2a
Kaempferitrin	5.24E+07	5.31E+07	4.64E+07	3.49E+07	3.10E+07	3.22E+07	-0.6	2b
sn-glycero-3-Phosphocholine	5.61E+06	5.08E+06	4.98E+06	2.20E+06	3.14E+06	2.03E+06	-2.4	2a
γ-Linolenic acid	5.20E+08	5.51E+08	5.30E+08	3.86E+08	3.68E+08	4.29E+08	0.3	1
N-Acetylornithine	1.88E+08	1.60E+08	1.80E+08	8.76E+07	1.11E+08	8.01E+07	-1.4	2b
5,5-Dimethylhydantoin	7.82E+07	7.58E+07	7.20E+07	4.17E+07	5.33E+07	4.01E+07	-0.7	2b
Choline	6.94E+09	7.47E+09	7.17E+09	5.97E+09	6.06E+09	5.63E+09	-0.4	2b
cGMP	1.24E+07	1.42E+07	1.09E+07	6.31E+06	6.55E+06	6.35E+06	-1.4	2a
Asparagine	3.18E+08	3.22E+08	2.90E+08	1.90E+08	2.15E+08	1.57E+08	-1.1	1
Glycine anhydride	2.56E+07	2.77E+07	2.51E+07	1.52E+07	1.79E+07	1.24E+07	-0.9	2b
Threonine	1.04E+08	1.13E+08	1.13E+08	7.50E+07	5.80E+07	5.00E+07	-0.5	1
1-Methyladenine	3.12E+07	3.48E+07	3.48E+07	1.49E+07	2.21E+07	1.99E+07	-0.5	2b
2-(3,4-Dihydroxyphenyl)-5-hydroxy-4-oxo-4H-chromen-7-yl 6-O-(6-deoxy-alpha-L-mannopyranosyl)-beta-D-glucopyranoside	1.20E+07	1.29E+07	1.24E+07	8.56E+06	8.83E+06	6.59E+06	-0.8	2b
UDP-N-acetylglucosamine	1.32E+05	1.24E+05	1.03E+05	5.03E+04	1.40E+04	2.20E+03	0.8	2b
Robinin	6.40E+06	6.29E+06	5.47E+06	4.49E+06	3.86E+06	3.74E+06	-0.5	2b
Kynurenic acid	1.52E+05	1.04E+05	1.35E+05	3.04E+04	3.38E+04	6.12E+04	-1.0	2a
Proline	5.87E+08	6.01E+08	5.14E+08	3.98E+08	4.35E+08	3.77E+08	-0.8	1
Methionine sulfoxide	3.14E+06	3.97E+06	3.73E+06	2.34E+06	1.65E+06	1.66E+06	-0.7	2a
Citrulline	3.10E+08	2.57E+08	2.60E+08	1.58E+08	1.97E+08	1.57E+08	-1.4	1

Table S3. List of the intensity levels, log2 ratio, and level of annotation of the statistically significant metabolites ($p<0.05$) found in the **roots** of WT *S. lycopersicum* cv. Micro-Tom after treatment with ^{12}C D-glucose vs. control for 48h under continuous illumination.

metabolite name	Control1	Control2	Control3	^{12}C Glc_1	^{12}C Glc_2	^{12}C Glc_3	log2ratio	Level of annotation
2-oxoglutaric acid	1.91E+08	1.71E+08	1.88E+08	3.39E+08	3.82E+08	3.17E+08	0.9	2a
N-acetylserine	1.12E+07	1.10E+07	6938106	2.37E+07	2.89E+07	2.89E+07	1.5	1
γ-aminobutyric acid	3.28E+08	3.44E+08	3.42E+08	1.04E+09	1.14E+09	9.36E+08	1.6	1
Succinic acid	8.18E+07	8.37E+07	8.84E+07	4.58E+08	5.10E+08	4.53E+08	2.5	1
Pyroglutamic acid	9.21E+07	5.01E+07	2.62E+07	3.83E+08	4.74E+08	4.23E+08	2.9	2a
Alanine	4.02E+07	3.38E+07	2.51E+07	2.75E+08	3.59E+08	3.40E+08	3.3	2a

Table S4. List of the intensity levels, log2 ratio, and level of annotation of the statistically significant metabolites ($p<0.05$) found in the **hypocotyl** of WT *S. lycopersicum* cv. Micro-Tom after treatment with ^{12}C D-glucose vs. control for 48h under continuous illumination.

metabolite name	Control1	Control2	Control3	^{12}C Glc_1	^{12}C Glc_2	^{12}C Glc_3	log2ratio	Level of annotation
Pyroglutamic acid	5.81E+07	3.97E+07	3.94E+07	4.41E+08	3.10E+08	4.37E+08	2.9	2a
Methylsuccinic acid	1.15E+07	1.73E+07	9169298	6.39E+07	5.88E+07	6.07E+07	2.7	2a
1,5-naphthalenediamine	1.35E+07	1.17E+07	1.47E+07	5.24E+07	5.19E+07	5.44E+07	1.1	2b
Tryptophan	1.68E+07	1.38E+07	1.56E+07	8.06E+07	6.01E+07	5.94E+07	0.4	1
Glutamine	8.57E+08	6.02E+08	5.41E+08	3.67E+09	3.25E+09	4.48E+09	0.2	1
5,5-Dimethylhydantoin	1.84E+07	1.37E+07	1.25E+07	1.05E+08	7.04E+07	8.89E+07	0	2b
Asparagine	1.22E+08	8.45E+07	6.14E+07	4.14E+08	3.08E+08	3.52E+08	-0.4	1
Tyrosine	2.08E+07	2.01E+07	1.16E+07	1.55E+08	1.22E+08	1.61E+08	-0.6	1
Diethanolamine	1.25E+08	1.18E+08	1.32E+08	1.06E+07	1.06E+07	1.80E+07	-3.5	2b

Table S5. List of identified metabolites with annotation levels of 1 and 2a and Pearson correlation ≥ 0.99 between roots and shoots of wild-type *A. thaliana* seedlings incubated with $^{13}C_6$ labeled Glc (30 mM) and kept under continuous illumination for 48h.

metabolite name	correlation	annotation level	potential attribution hexoses
Fructose 6-phosphate	0.9983	1	
Glycine	1.0000	2a	
GMP (Guanosine monophosphate)	0.9988	2a	
Hexose	0.9945	1	tagatose
Hexose dimer2	0.9970	1	melibiose
Hexose dimer3	1.0000	1	cellobiose/maltose
Hexose1	0.9999	1	glucose/galactose
Hexose2	1.0000	2a	inositol species
Indole-3-carboxylate	0.9987	1	
Isethionic acid	1.0000	2a	
Isoleucine	0.9996	1	
Isonicotinic acid	1.0000	2a	
Lactic acid	0.9997	2a	
Methionine	0.9997	2a	
Methionine sulfoxide	1.0000	2a	
N-Acetyl-arginine	0.9999	2a	
N-Acetyl-hexoseamine1	1.0000	2a	
Pinitol	0.9956	2a	
Pipecolinic acid	0.9999	1	
Proline	0.9953	1	
Pyroglutamic acid	0.9990	2a	
Pyruvic acid	1.0000	2a	
Taurine	1.0000	2a	
Tryptamine	1.0000	1	

Table S6. List of Identified metabolites with annotation levels of 1 and 2a and Pearson correlation ≥ 0.99 between **root and hypocotyl** of wild-type *S. lycopersicum cv. Micro-Tom* seedlings incubated with $^{13}C_6$ labeled Glc (30 mM) and kept under continuous illumination for 48h.

metabolite name	correlation	annotation level	potential attribution hexoses
Ferulic acid	0.9979	1	
Glucose-6-phosphate	0.9953	1	
Glycerol 3-phosphate	0.9998	1	
Hexose dimer2	0.9914	1	melibiose
Cinnamic acid	0.9997	2a	
Pinitol	0.9954	2a	
sn-glycero-3-phosphocholine	0.9938	2a	
Taurine	1.0000	2a	
Uridine monophosphate (UMP)	0.9979	2a	

Table S7. List of identified metabolites with annotation levels of 1 and 2a and Pearson correlation ≥ 0.99 between **hypocotyl and leaf** of wild-type *S. lycopersicum cv. Micro-Tom* seedlings incubated with $^{13}C_6$ labeled Glc (30 mM) and kept under continuous illumination for 48h.

metabolite name	correlation	annotation level
Aldopentose	0.9951	1
Cinnamic acid	1.0000	1
Indole-3-carboxylate	0.9970	1
Isoleucine	0.9968	1
Pinitol	1.0000	1
Serine	0.9975	1
Citraconic acid	1.0000	2a
Glycine	1.0000	2a
Methionine	0.9949	2a
Pentose-5-phosphate	0.9989	2a
Phosphoenolpyruvic acid	1.0000	2a

Table S8. List of identified metabolites with annotation levels of 1 and 2a and Pearson correlation ≥ 0.99 between **root and hypocotyl** of *G. hirsutum* seedlings incubated with $^{13}C_6$ labeled Glc (30 mM) and kept under continuous illumination for 48h.

metabolite name	correlation	annotation level	potential attribution hexoses
Hexose dimer2	1.0000	1	melibiose
Hexose dimer1	0.9934	1	?
Cinnamic acid	1.0000	2a	
Taurine	1.0000	2a	
N-Acetyl-hexoseamine1	1.0000	2a	
GMP	1.0000	2a	
4-pyridoxic acid	0.9999	2a	
5-methoxysalicylic acid	0.9996	2a	

Table S9. List of identified metabolites with annotation levels of 1 and 2a and Pearson correlation ≥ 0.99 between **hypocotyl** and **first true leaves** of *G. hirsutum* seedlings incubated with $^{13}C_6$ labeled Glc (30 mM) and kept under continuous illumination for 48h.

metabolite name	correlation	annotation level	potential attribution hexoses
Hexose2	1.0000	1	glucose/galactose
Glyceric acid	1.0000	1	
Trigonelline	0.9996	1	
Adenine	0.9996	1	
Hexose dimer1	0.9990	1	?
Serine	0.9989	1	
Aldopentose	0.9934	1	
Cinnamic acid	1.0000	2a	
Indole	1.0000	2a	
Glycine	1.0000	2a	
CMP (Cytidine monophosphate)	1.0000	2a	
N-Acetyl-hexoseamine1	1.0000	2a	
Lactic acid	1.0000	2a	
Fructose-1-phosphate	0.9995	2a	
Ethylmalonic acid/Methylsuccinic acid	0.9988	2a	
Pyruvic acid	0.9986	2a	
Aspartic acid	0.9950	2a	
Methionine	0.9936	2a	
Nicotinamide	0.9914	2a	

Table S10. List of identified metabolites with annotation levels of 1 and 2a and Pearson correlation ≥ 0.99 between tissues and plant species.

**WT *Micro-Tom* root-hypocotyl**	***G. hirsutum* root-hypocotyl**	**WT *A. thaliana* root-shoot**		**WT *Micro-Tom* hypocotyl-leaves**	***G. hirsutum* hypocotyl-leaves**	**WT *A. thaliana* root to shoot**
Ferulic acid	Hexose dimer2	Fructose 6-phosphate		Aldopentose	Hexose2	Fructose 6-phosphate
Glucose-6-phosphate	Hexose dimer1	Glycine		Cinnamic acid	Glyceric acid	Glycine
Glycerol 3-phosphate	Cinnamic acid	GMP		Indole-3-carboxylate	Trigonelline	GMP
Hexose dimer2	Taurine	Hexose		Isoleucine	Adenine	Hexose
Cinnamic acid	N-Acetyl-hexoseamine1	Hexose dimer2		Pinitol	Hexose dimer1	Hexose dimer2
Pinitol	GMP	Hexose dimer3		Serine	Serine	Hexose dimer3
sn-glycero-3-Phosphocholine	4-pyridoxic acid	Hexose1		Citraconic acid	Aldopentose	Hexose1
Taurine	5-methoxysalicylic acid	Hexose2		Glycine	Cinnamic acid	Hexose2
UMP		Indole-3-carboxylate		Methionine	Indole	Indole-3-carboxylate
		Isethionic acid		Pentose-5-phosphate	Glycine	Isethionic acid
		Isoleucine		Phosphoenolpyruvic acid	CMP (Cytidine monophosphate)	Isoleucine
		Isonicotinic acid			N-Acetyl-hexoseamine1	Isonicotinic acid
		Lactic acid			Lactic acid	Lactic acid
		Methionine			Fructose-1-phosphate	Methionine
		Methionine sulfoxide			Ethylmalonic acid/Methylsuccinic acid	Methionine sulfoxide
		N-Acetyl-arginine			Pyruvic acid	N-Acetyl-arginine
		N-Acetyl-hexoseamine 1			Aspartic acid	N-Acetyl-hexoseamine1
		Pinitol			Methionine	Pinitol
		Pipecolinic acid			Nicotinamide	Pipecolinic acid
		Proline				Proline
		Pyroglutamic acid				Pyroglutamic acid
		Pyruvic acid				Pyruvic acid
		Taurine				Taurine
		Tryptamine				Tryptamine
